# Promiscuous Expression of H2B-GFP Transgene in Hematopoietic Stem Cells

**DOI:** 10.1371/journal.pone.0002357

**Published:** 2008-06-04

**Authors:** Grant A. Challen, Margaret A. Goodell

**Affiliations:** 1 Center For Cell and Gene Therapy, Baylor College of Medicine, Houston, Texas, United States of America; 2 Monash University, Clayton, Victoria, Australia; City of Hope Medical Center, United States of America; City of Hope Medical Center and Beckman Research Institute, United States of America

## Abstract

**Background:**

The study of adult stem cells relies on the ability to isolate them using complex combinations of markers for flow cytometry. A recent study has used a tetracycline-regulatable H2B-GFP transgenic mouse model analogous to BrdU pulse-chase methods to fluorescently label quiescent skin stem cells in vivo. In this study, we sought to use these mice to fluorescently label hematopoietic stem cells to study niche interactions.

**Methods and Findings:**

We crossed the H2B-GFP mice to mice carrying a tetracycline-regulated transactivator protein. When these mice were administered doxycycline, we observed a gradual decrease in total bone marrow GFP^+^ cells over 12 weeks but the hematopoietic stem cell population remained largely GFP^+^ (>85%). In histological bone sections, the long-term GFP label-retaining cells tended to concentrate at the endosteal surface and competitive transplantation assays showed that the majority of hematopoietic stem cell activity was contained in the GFP^+^ cell fraction. However, in response to stimulation with 5-fluorouracil, the hematopoietic stem cells of the crossed mice still retained a high level of GFP expression when it was anticipated the label should be lost when the cells divide. Upon further review, it was determined that the founder H2B-GFP mice showed spurious expression of the transgene at high levels in the hematopoietic stem cell population, thus the observed response of hematopoietic stem cells in the double transgenic mice to doxycycline was due to aberrant expression of the transgene and not the correct tetracycline-regulatable system.

**Conclusions:**

We observed promiscuous expression of the H2B-GFP transgene in the hematopoietic stem cell compartment of the bone marrow. This leaky expression prohibits the use of this model to study hematopoietic stem cells in vivo and careful characterization for each organ must be done if this transgenic system is to be used to isolate other prospective tissue stem cells.

## Introduction

Adult stem cells are characterized by the ability to self-renew to replenish the stem cell pool and the capacity to differentiate and generate specialized cell types of their tissue of residence to replace damaged or lost cells. The study of adult stem cells relies on the ability to identify and isolate them from the tissue, with the latter usually requiring multi-color flow cytometry harnessing a combination of positive and/or negative cell surface markers which distinguish the stem cell from other cells of the tissue. For example, mouse hematopoietic stem cells (HSCs) are typically isolated using some combination of Sca-1^+^, c-kit^+^, CD150^+^ [1] and lack of expression of markers of mature hematopoietic cells types such as CD4, CD8, Gr-1, Mac-1, Ter119 and B220 (lineage negative or Lin^−^). Such complex marker combinations make the *in vivo* study of stem cells in their niche difficult by immuno-histology.

Another general characteristic of adult stem cells is the state of quiescence or slow cell turnover under normal physiological conditions. In general, stem cells have a slow rate of turnover at steady state to conserve growth potential and prevent genetic injury during mitosis. This property has been used to identify putative stem cells in skin [2], kidney [3], cornea [4] and prostate [5] by BrdU pulse-chase experiments. Recently, a group developed a transgenic mouse methodology to fluorescently label slow cycling cells such as prospective stem cells. The transgenic mice express histone H2B-GFP controlled by a tetracycline-responsive element (TRE) upstream of a CMV promoter [6]. The H2B-GFP expression in these mice should be silent in the absence of a tetracycline transactivator protein (tTA). When the H2B-GFP mice are crossed to mice harboring a promoter-driven tetracycline-regulatable transactivator protein, H2B-GFP fluorescence is produced when the tTA binds to the TRE. The H2B–GFP fusion protein becomes incorporated into nucleosomes without affecting cell cycle progression [7]. The H2B-GFP fusion protein makes the fluorescent label very stable and the nuclear localization also facilitates easier identification of label-retaining cells in histological sections. When treated with doxycycline, the tTA complex is released from the TRE and GFP expression is lost when the cells divide and H2B-GFP is replaced in nucleosomes by normal unlabelled histone 2B. Hence only the non-dividing cell population will retain H2B-GFP expression over a long period of time in the presence of doxycycline.

The group that generated the H2B-GFP mice used a keratin 5 promoter-driven tetracycline-regulated transactivator to specifically label the skin [6]. After a 4-week doxycycline chase, the only GFP^+^ cells were in the bulge, the presumptive skin stem cell compartment [8]. Although the original authors used these mice to study skin stem cells, these H2B-GFP mice could conceivably be crossed with mice expressing other tissue-specific promoter-driven tetracycline-regulatable transactivators to isolate quiescent GFP^+^ label-retaining cells (presumptive tissue stem cells) from other organs where resident stem cells have not been isolated or for which no markers for isolation exist such as pancreas and kidney. Moreover, labeling stem cells *in vivo* with a single tag (GFP) would allow the study of stem cell-niche interactions with more complex immunofluorescent multi-marker staining. This aspect of stem cell biology has been hampered in the hematopoietic field by virtue of the multitude of markers required to identify such a numerically minor cell population of the bone marrow (HSCs are typically found in the bone marrow at a frequency of 0.01%). Although one study recently used a somewhat simplified combination of markers (CD150^+^CD48^−^CD41^−^Lin^−^) to identify HSCs in histological sections [9], this strategy still required combining multiple antigens with the same fluorophore and used all available spectral channels. Even using this strategy leaves few if any available spectral channels to label sections with markers of endothelial cells, osteoblasts or bone marrow stroma in combination with HSCs to study the hematopoietic stem cell niche *in vivo*. One of the major advantages to using this H2B-GFP transgenic model is the potential ability to label quiescent stem cells with a single fluorescent marker (GFP) thereby freeing up all other spectral channels for co-staining with markers of other cell types found in the HSC niche.

**Figure 1:**
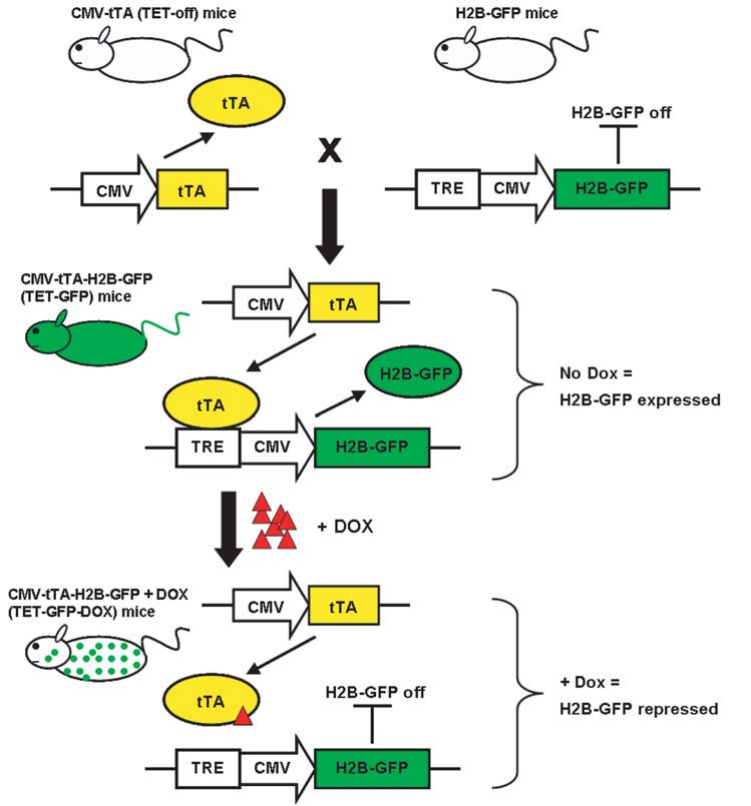
Schematic representation of transgenic mouse breeding scheme. H2B-GFP mice should not express GFP in the absence of a tetracycline-regulatable transactivator. When these mice are crossed to mice expressing tTA from a CMV promoter (TET-off mice), the resulting mice (TET-GFP) express GFP when the tTA binds to the tetracycline responsive element (TRE). When these mice are chased with doxycycline (TET-GFP-DOX) the doxycycline binds to the tTA complex, dislodging it from the TRE and turning off expression of GFP. GFP expression is lost once the cells divide therefore only the quiescent cells retain the GFP label long-term.

Because there is no one marker that is uniquely and specifically expressed by HSCs, we sought to use the H2B-GFP mice to fluorescently label HSCs *in vivo*. In the absence of a bone marrow-specific promoter-driven tetracycline-regulatable transactivator mouse strain, we crossed H2B-GFP mice to transgenic mice expressing tTA from a generic CMV promoter (CMV-tTA mice, referred to from here as TET-off mice since the addition of doxycycline results in repression of genes under the control of a TRE). When the progeny of this cross (CMV-tTA-H2B-GFP mice, referred to from here as TET-GFP mice; Figure 1) were administered doxycycline in their drinking water, we observed a decrease in total bone marrow GFP^+^ cells over a 12 week treatment timecourse while approximately 90% of the HSC population remained GFP^+^ after this time. Histologically, GFP^+^ cells were most concentrated at the endosteal surface, one of the proposed HSC niches [10]. Because of the potential power of this tetracycline-regulatable system we carefully analyzed all of the parameters before undertaking a large study. Upon further investigation, it was discovered that the H2B-GFP founder mice showed leaky expression of GFP in the bone marrow, and in particular spurious and high expression of the transgene in the HSC compartment. These mice should not show any H2B-GFP expression prior to being crossed to the TET-off mice because no tTA protein is present to drive GFP expression from the TRE. This leaky background GFP expression in the bone marrow of the uncrossed H2B-GFP mice makes them unsuitable for the purposes of labeling HSCs *in vivo*. However, the observation that the H2B-GFP transgene is expressed almost constitutively by the HSC population is interesting and may warrant further investigation into the mechanism.

## Results

### Bone Marrow Response of TET-GFP Mice to Doxycycline

**Figure 2:**
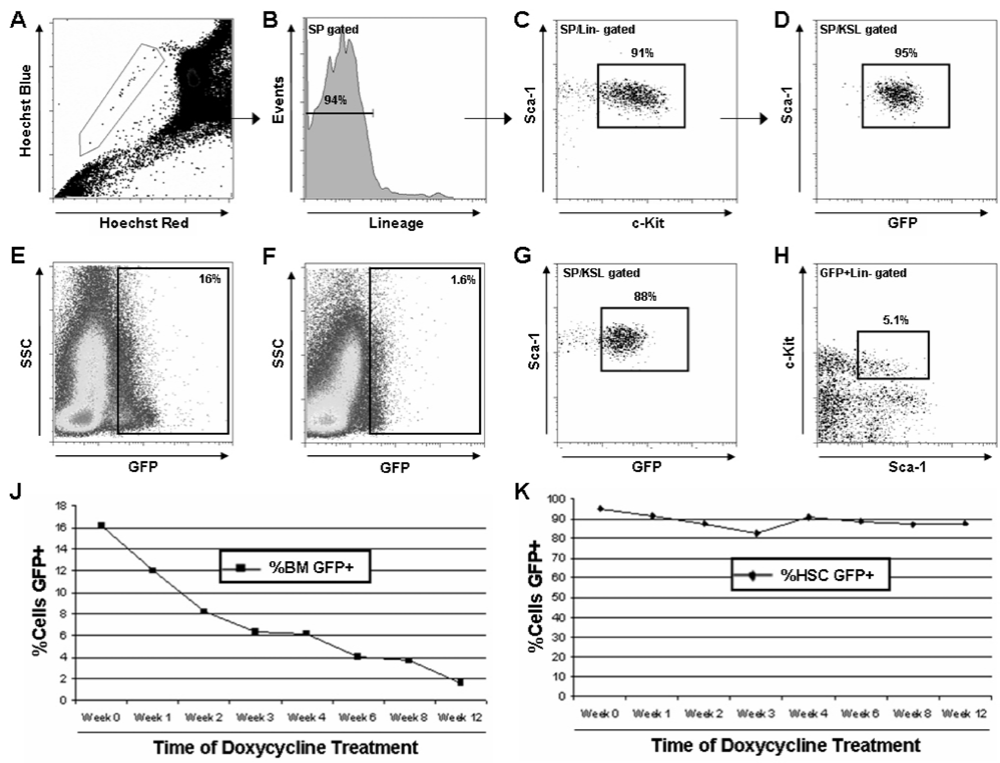
Flow cytometric analysis. (A–D) Gating scheme used to identify mouse hematopoietic stem cells which were defined as side population (A), lineage negative (B), c-Kit^+^Sca-1^+^ (C). This population was then gated to a Sca-1/GFP plot to identify the proportion of HSCs that were GFP^+^. (D) HSCs from TET-GFP mice prior to administration of doxycycline were 95% GFP^+^. (E–F) Proportion of GFP^+^ cells in whole bone marrow of TET-GFP mice prior to dox treatment (E) and after 12 weeks of dox treatment (F). The percentage of GFP^+^ cells in whole bone marrow decreased in response to dox. (G) HSCs from TET-GFP mice after 12 weeks treatment with dox were still over 85% GFP^+^. (H) However, after 12 weeks of dox treatment not all GFP^+^ cells from TET-GFP mice were HSCs as only 5% of GFP^+^Lin^−^ cells were Sca-1^+^c-Kit^+^. The percentage of GFP^+^ whole bone marrow cells from TET-GFP mice decreased in response to dox (J) while the majority of HSCs remained GFP^+^ (K).

In the system presented in this study, TET-GFP mice should show a reduction in GFP^+^ cells in response to continuous doxycycline (dox) treatment as the dox causes the tTA to be released from the TRE and H2B-GFP expression is lost. H2B-GFP is replaced by normal H2B when the cells divide and hence only the non-dividing cells (in this case theoretically HSCs) retain the GFP label long-term. The TET-GFP mice were treated with a dox timecourse to determine the proportion of the GFP^+^ cells in the bone marrow and HSC compartment over time (Figure 2). Prior to administration of dox, the GFP^+^ fraction of the total bone marrow ranged from 15–20% (average 16.2%) which was lower than anticipated when crossing to a generic CMV promoter-driven tetracycline-regulatable transactivator, although this may be a function of having both the tTA and H2B-GFP driven by separate CMV promoters in the same cell. Also the GFP signal was quite dim when analyzed by flow cytometry although the GFP^+^ population could confidently be identified when compared to wild type control animals. Regardless, the HSC population of these mice prior to dox treatment was close to 95% GFP^+^. Every week for 12 weeks of dox treatment, two mice were taken for flow cytometry analysis (Table 1). As expected, the proportion of GFP^+^ cells in the total bone marrow, comprised of cells that are rapidly turned over, decreased gradually to ∼1.6% at 12 weeks while the proportion of GFP+ cells in the slowly-dividing HSC population declined only slightly, dropping to only ∼85%. The slight fluctuations in HSC GFP^+^ percentage between time-points can be attributed to variation between individual animals.

**Table 1:** Proportion of GFP^+^ cells in whole bone marrow and hematopoietic stem cell population from TET-GFP mice treated with doxycycline.

	Week 0	Week 1	Week 2	Week 3	Week 4	Week 6	Week 8	Week 12
**%BM GFP+**	16.2	12.0	8.2	6.4	6.2	4.1	3.7	1.6
**%HSC GFP+**	94.9	91.4	87.7	82.7	90.6	88.5	87.1	87.6

It should be noted that although this initial analysis appeared successful in labeling the HSC population, there was still some element of background GFP expression in other cell types even at these long time-points. This could be demonstrated by the fact that only about 5% of GFP^+^Lin^−^ cells from TET-GFP mice after 12 weeks of dox treatment also expressed the stem cell markers Sca-1^+^c-Kit^+^ indicating that many non-stem cells also retained GFP expression. This presented an important caveat in that although almost all HSCs remained GFP^+^, not all GFP^+^ cells were HSCs.

### Localization of GFP^+^ Cells *in situ*

**Figure 3:**
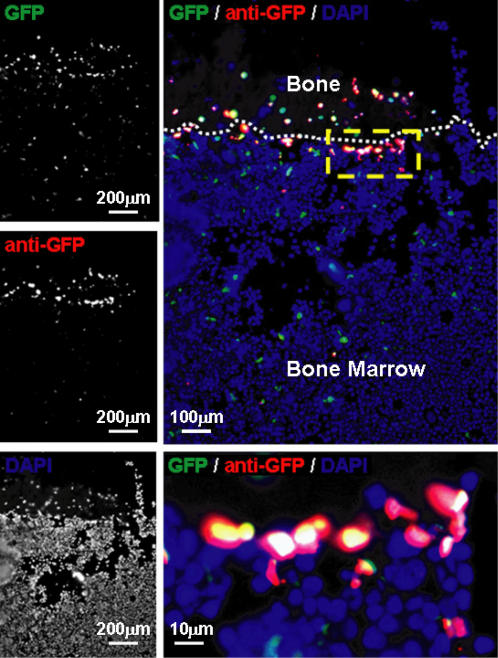
Bone section of a TET-GFP mouse treated with doxycycline for 12 weeks. An anti-GFP antibody was used in combination to correctly identify GFP^+^ cells due to autofluorescence in the sections. In the merged image, the white dashed line indicates the endosteal surface at the bone/bone marrow interface. Concentrations of GFP^+^/anti-GFP^+^ cells from TET-GFP were witnessed at the endosteal surface, one of the proposed HSC niches. The yellow box in the merged image is magnified below to more clearly show a cluster of double positive cells.

One of the major purposes of using this fluorescent labeling strategy was to facilitate the study of HSCs with their niche *in vivo*. After 12 weeks of dox treatment, the femurs of TET-GFP mice were fixed and sectioned to localize the GFP^+^ cells. Because we observed a high level of background autofluorescence in bone sections of wild-type controls, an anti-GFP antibody was used in conjunction with H2B-GFP fluorescence to confidently identify the labeled cells of TET-GFP mice (autofluorescent signals tended to be restricted to either red or green channels) . While double positive cells (GFP^+^/anti-GFP^+^) were observed scattered throughout the bone marrow (Figure 3), we did find concentrations of these cells at the endosteal surface of the bone interfacing with the marrow cavity. The endosteal surface has been proposed as a predominant HSC niche by numerous studies [10]. However, as discussed above, despite the fact that almost all phenotypically-defined HSCs were GFP^+^ at this timepoint, not all GFP^+^ cells were HSCs. Thus to definitively imply that the GFP^+^ cells in these sections were HSCs, other additional markers would need to be used (CD150, Sca-1, c-Kit) in combination, a task made difficult by the lack of filters available for epifluorescence microscopy. In keeping with the fact that GFP is not necessarily a good marker of HSCs in the TET-GFP mice treated with dox, we also found many GFP^+^/anti-GFP^+^ cells scattered throughout the bone marrow in these sections in areas not necessarily associated with a HSC niche.

### Competitive Bone Marrow Transplantation

**Figure 4:**
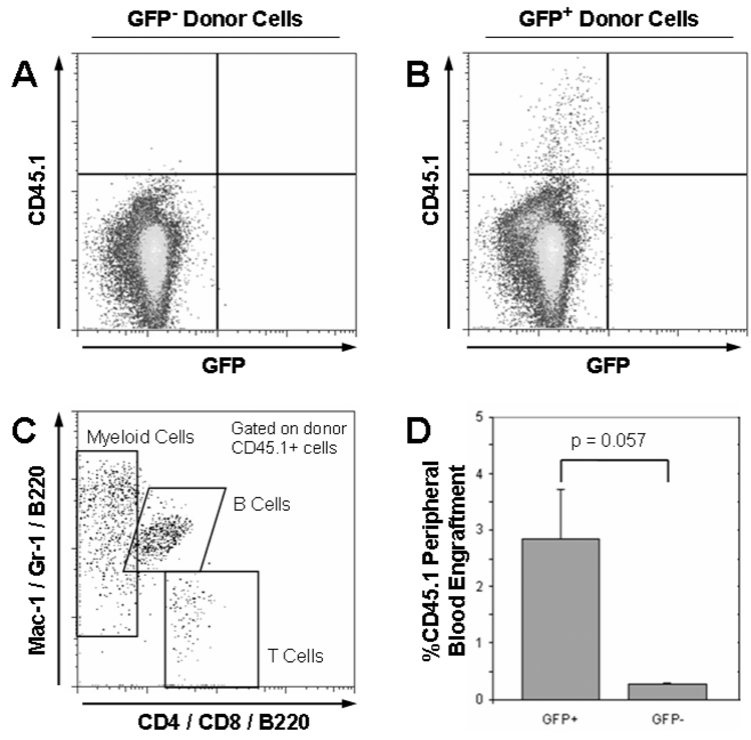
Competitive transplantation of GFP^+^ and GFP^−^ cells from TET-GFP mice treated with doxycycline for 12 weeks. Analysis of peripheral blood of mice transplanted with GFP^−^ (A) and GFP^+^ (B) cells 12 weeks post-transplant showed that only cells from the GFP^+^ fraction were able to contribute to hematopoiesis of recipient mice. (C) Gating of donor CD45.1 cells from GFP^+^ cell transplant recipients showed that they could contribute to all major lineages of the peripheral blood (B cells, T cells, myeloid cells). (D) Average peripheral blood engraftment of mice transplanted with either GFP^+^ or GFP^−^ cells from TET-GFP mice treated with doxycycline for 12 weeks (n = 4). GFP^+^ donor cells showed significantly higher engraftment levels with any engraftment of GFP^−^ donor cells being likely artefactual.

To test HSC function, GFP^+^ and GFP^−^ cells were purified from the bone marrow of TET-GFP mice treated with doxycycline for 12 weeks. No other stem cell markers were in this isolation, which relied on GFP alone to serve as a marker of HSCs. 200 GFP^+^ or GFP^−^ donor cells (CD45.1) were competitively transplanted into lethally irradiated CD45.2 recipient mice (n = 4) along with 200,000 CD45.2 whole bone marrow competitor cells. 12 weeks post-transplant, peripheral blood was analyzed for donor cell engraftment, GFP fluorescence and lineage contribution (Figure 4). All of the HSC activity was located in the GFP^+^ fraction, although the chimerism of recipients blood was low (2.84 ± 0.87%). This peripheral blood engraftment was significantly higher (p = 0.057) than mice transplanted with GFP^−^ cells (0.27 ± 0.05%), in which donor cells were barely detectable compared to background staining levels. Donor GFP^+^ cells were seen to have multi-lineage potential with robust contribution seen to myeloid, B cell and T cell compartments. However, the low level of engraftment suggested that although the GFP^+^ cell fraction was enriched for HSC activity compared to the GFP- fraction, it still was not a pure HSC population, an observation consistent with our previous findings. Another caveat was that the progeny of donor GFP^+^ cells in the peripheral blood were GFP^−^ after 12 weeks, which was anticipated as GFP expression should be lost when the GFP^+^ cells divide.

### Response to 5-FU

**Figure 5:**
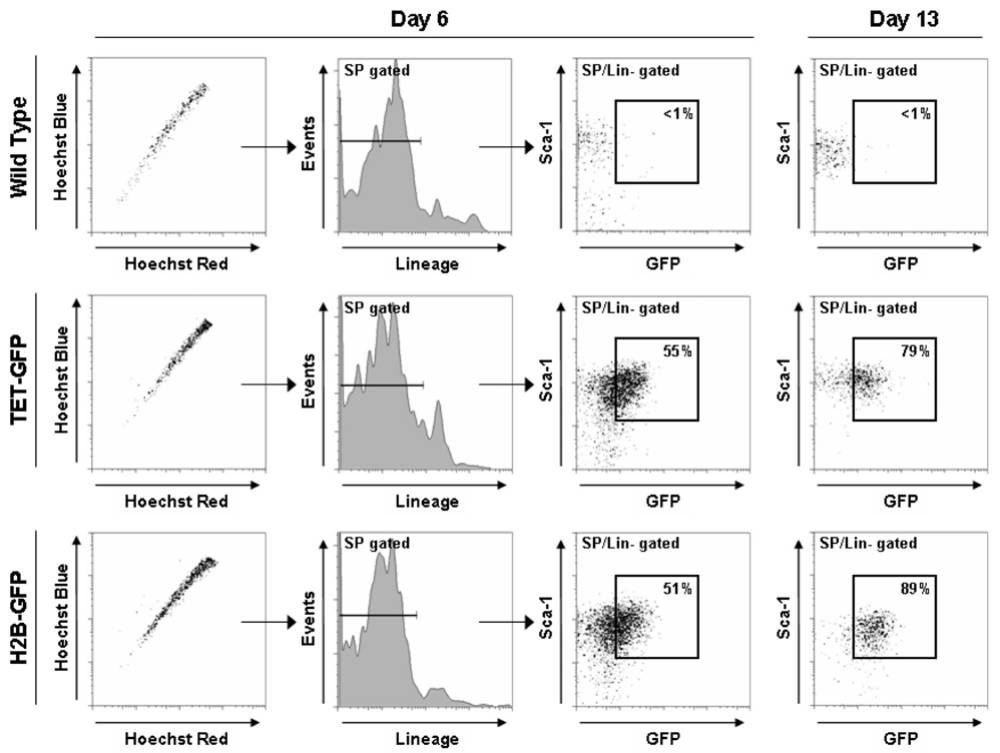
Hematopoietic stem cell response of wild-type, dox-treated TET-GFP and H2B-GFP mice to 5-FU. The HSC response of TET-GFP mice + dox (middle panels) and H2B-GFP mice lacking the tetracycline-regulated tTA (bottom panels) are identical. Note in particular the level of GFP expression at day 13 in all three genotypes (far right column), the proportion of GFP^+^ HSCs actually increased in TET-GFP mice +dox and H2B-GFP from day 6 to day 13.

We then attempted to determine if the long-term dox-treated GFP-labeled cells of TET-GFP mice could respond appropriately to a given stimulus. A single injection of the pyrimidine analog 5-FU kills cycling hematopoietic cells and forces the quiescent HSC population into cycle to repopulate the ablated hematopoietic system. HSC proliferation proceeds in a time-dependent manner [11], peaking 5–6 days after injections (in this experiment HSCs were defined as SP^+^Lin^−^Sca-1^+^ as c-Kit is downregulated on these cells in response to 5-FU). Therefore, upon stimulation by 5-FU, the quiescent GFP-labeled HSC population should be forced into cycle and GFP expression would be lost as the cells divide. 6 days after injection of 5-FU, approximately 55% of HSCs from TET-GFP mice were still GFP^+^, while 13 days after injection the proportion of GFP^+^ HSCs actually increased back up to ∼80% (Figure 5). This result was surprising, as it was expected that some HSCs would remain in quiescence and hence retain the GFP^+^ label, but the majority of HSCs should be stimulated to proliferate to replace the ablated hematopoietic system and hence would lose their GFP upon cell division. Moreover, the proportion of GFP^+^ cells actually increased from day 6 to day 13 after 5-FU. This should not occur when these mice are continuously treated with dox , because once the GFP is lost, it should never be able to be regained as long as dox is present, and hence the proportion of GFP^+^ HSCs should only continue to decrease.

**Figure 6:**
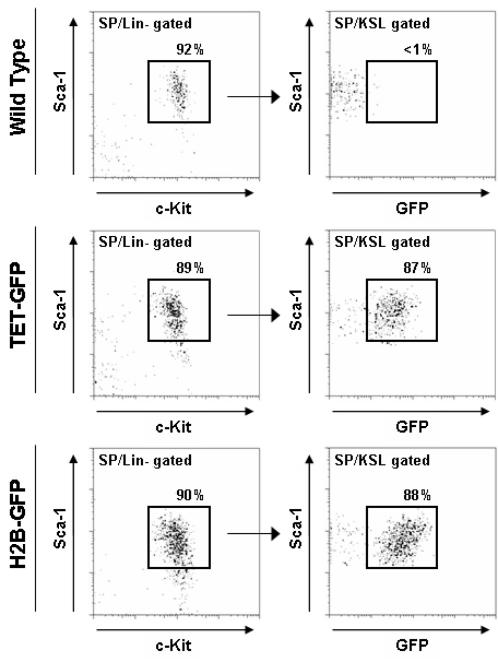
Hematopoietic stem cells of wild-type, dox-treated TET-GFP and uncrossed H2B-GFP mice lacking the tetracycline-regulated transactivator. The HSCs of H2B-GFP mice show strong expression of GFP even in the absence of the tTA.

This result, and the observation that such a high proportion of HSCs were still GFP^+^ after the 12-week dox treatment, conflicted with data regarding the proportion of HSCs actively proliferating and cycling at any given point in time [12]. We went back to analyze the H2B-GFP mice in the absence of the tet-transactivator allele using the same assays (Figure 6). Even though the H2B-GFP mice express no tetracycline-regulated transactivator, and thus should not express GFP, we found that the HSCs of this mouse strain were in fact >85% GFP^+^. In addition, it should be noted that even in peripheral blood and whole bone marrow, we could detect a low level of GFP expression. Therefore this strain displays promiscuous expression of H2B-GFP in certain hematopoietic cell types, but in particular the HSC population. Although it did appear that the majority of bone marrow from TET-GFP mice did respond appropriately in response to doxycycline (gradual reduction in GFP^+^ cells over time), GFP^+^ cells could still be detected in whole bone marrow at a frequency of about 1.6% after 12 weeks of dox. Although it was anticipated that some quiescent HSCs, would still be labeled after this length of time, the majority of GFP^+^ cells must be attributed to leaky expression of the transgene from the H2B-GFP strain. Of particular note, the transgene appeared to be extremely active in HSCs. For example, in the TET-GFP mice after 12 weeks dox treatment, approximately 3 out of every 200 bone marrow cells were GFP^+^, but from the same animal, approximately 170 out of every 200 HSCs were GFP^+^. Although GFP^+^ cells were enriched in the HSCs, not all GFP^+^ cells were HSCs, as the frequency of HSCs in bone marrow is about 0.01% compared to a frequency of GFP^+^ cell occurrence at 1.6%. Moreover, the HSC response of the H2B-GFP mice to 5-FU was exactly the same as the dox-treated TET-GFP mice. Taken together, these results indicate that any HSC response in the TET-GFP mice was not due to the appropriate tetracycline-regulated system, but rather spurious expression of the transgene specifically in the HSC population.

## Discussion

The ability to fluorescently label a quiescent (potential stem cell) population *in vivo* using a transgenic tetracycline-regulatable system is potentially a very powerful tool for the study of adult stem cells. It presents many advantages over the traditional BrdU pulse-chase methodology such as the ability to isolate viable long-term label-retaining cells by flow cytometry for *in vitro* or transplantation assays. The group that developed the system originally used a very elegant system to identify the bulge cells of the skin as the quiescent GFP label-retaining population in this organ [6] and these cells have subsequently been characterized as skin stem cells [8].

We sought to use a similar model to fluorescently label HSCs *in vivo* to study niche interactions in the bone marrow. While our initial results were encouraging in that we appeared to retain GFP label primarily in the HSC population in response to long-term doxycycline treatment, labeled cells appeared to localize to the endosteal niche in bone sections, and competitive transplantation showed that functional HSC activity was restricted to the GFP^+^ cell fraction, it was later discovered that retention of GFP expression in the double transgenic TET-GFP mice was in fact due to tet-transactivator-independent expression of the H2B-GFP mouse transgene. Notably, the transgene was especially activated in the HSC population, which made any results obtained from the dox-treated TET-GFP mice uninterpretable.

One could argue that the H2B-GFP mice themselves might be useful to study HSCs *in vivo* because almost all of the HSCs are GFP^+^. But these mice also show expression of GFP in other bone marrow cells (in fact these mice were routinely genotypyed by flow cytometry of the peripheral blood where we observed that ∼5% of the nucleated blood cells were GFP^+^), so while almost all HSCs are GFP^+^ not every GFP^+^ cell is a HSC. We observed an average of 1.8% of GFP^+^ cells in whole bone marrow from these animals (data not shown), which is almost identical to the proportion of GFP^+^ cells in the bone marrow of TET-GFP mice treated with dox for 12 weeks (1.6%). This again suggests that the long-term GFP-labeled cells from the TET-GFP mice were due to a leaky background expression from the H2B-GFP strain and not the appropriate tetracycline-regulated response. The H2B-GFP transgene was particularly active in the HSC compartment of the H2B-GFP mice, and in fact, if GFP was used as the only marker for purification of cells from the bone marrow, it would enrich for HSC activity approximately 48-fold compared to whole bone marrow (given 1.8% of whole bone marrow cells are GFP^+^, 85% of HSCs are GFP^+^ and assuming a normal HSC frequency in bone marrow of ∼0.01%). To use the GFP^+^ HSCs from the H2B-GFP mice to localize HSC within their bone marrow niche, the problem of leaky H2B-GFP expression in non-HSCs could potentially be circumvented by purifying HSCs from these mice and transplanting them into a wild-type recipient. But the HSCs are then exposed to a strong selective pressure and may not home to their normal niche or behave in their typical fashion. This also may not solve the problem since the transplanted HSCs will generate progenitors which may still express the transgene. Also one of the aims of using this system was to locate HSCs *in vivo* by labeling with only one color (GFP) for immunofluorescence analysis. Labeling HSCs with one color would facilitate the use of other markers to study HSC-niche interactions with endothelial cells, osteoblasts or other cell types. The fact that not all GFP^+^ cells were HSCs in H2B-GFP and TET-GFP mice treated with dox for 12 weeks again limits the usefulness of this system as multiple markers (i.e. Sca-1, c-kit, CD150) would need to be used to correctly identify HSCs thereby limiting the number of available colors for other cell types.

The reason for this spurious GFP expression specifically in the HSC population is unclear, but somewhat interesting. The vast majority of bone marrow cell types showed a tight regulation of GFP in response to dox treatment which poses the question of why the HSC population relatively specifically showed aberrant GFP expression in the absence of the tTA protein. Perhaps the transgene was integrated into a genomic region that is permissive for expression in HSCs or a locus that exhibits high transcriptional activity. Another explanation could be that, as suggested by recent studies, HSCs and other stem cells have a more “open” chromatin structure to facilitate rapid gene transcription for multiple cell lineage differentiation programs with chromatin becoming more closed as differentiation occurs [13]. This could make tetracycline-regulated transgene expression not as tight in HSCs compared to differentiated cells which have a more rigidly controlled transcriptional program. In response to 5-FU, the proportion of GFP^+^ HSCs from H2B-GFP and TET-GFP mice treated with dox decreased from 85% at day 0 to ∼55% at day 6, but then increased back up to ∼80% by day 13. So there was some response of the transgene in the HSCs of these animals. These results may suggest that the H2B-GFP transgene is integrated in a locus that becomes transcriptionally repressed during HSC activation and proliferation (day 6 post 5-FU), but then resumes transcriptional activity upon return of HSCs to a more stable, quiescent state (day 13 post 5-FU). An alternative explanation for this may be that for some reason, the GFP^−^ HSCs might have died or differentiated such that the GFP^+^ cells became a higher proportion of the total HSC population.

Another possibility is that H2B-GFP fusion protein itself affects cell behavior. Although the fusion protein enables sensitive analysis of chromosome dynamics in living mammalian cells *in vitro* [7], there is little evidence to suggest it has no impact on cell function *in vivo*. The original study using this methodology to label skin stem cells did not show any adverse effects from the H2B-GFP transgene [6], it has not been conclusively shown that H2B-GFP does not affect the gene expression profile or cell fate decisions in various cell populations, including HSCs. The chimeric H2B-GFP protein is composed of the 126 amino acid H2B histone fused to the 239 amino acid GFP via a six amino acid linker. The presence of the GFP moiety, which is almost twice the molecular weight of H2B, makes the chimeric H2B-GFP much bulkier than the wild type histone protein and it is possible that the presence of this extra protein in nucleosome core particles does affect the fine structure of the chromatin. The incorporation of H2B-GFP into chromatin may affect gene expression profile and cell fate commitments via multiple mechanisms including effects on nucleosome structure, chromatin structure, histone code, epigenetic regulation of gene expression, asymmetric cell division and potential “leakiness” of the doxycycline-inducible system itself.

Chromatin structure has been shown to be crucial to normal HSC function and any disruption to the normal architecture by inclusion of H2B-GFP could have deleterious effects. While the inner core of the nucleosome is very stable, it has been shown H2B on the surface of active nucleosomes exchanges continually [14], thus permitting cell lineage visualization by H2B-GFP in transgenic animals, but also allowing for the possible perturbation of HSC function by replacement of wild type H2B with H2B-GFP. Furthermore, ubiquitination of H2B affects the methylation status of H3 [15], thus substitution of H2B-GFP for wild type H2B may alter the epigenetic histone code in these transgenic animals. Epigenetic modifications have been shown to serve as an important mechanism that controls HSC multipotency and cell fate decisions and several observations suggest that epigenetic modifications (including histone modifications) are present at lineage gene-specific loci in HSCs [16]. The molecular processes governing hematopoiesis involve the interplay between lineage-specific transcription factors and a series of epigenetic tags, including DNA methylation and covalent histone tail modifications such as acetylation, methylation phosphorylation, SUMOylation and ubiquitylation. These post-transcriptional modifications, which collectively constitute the “histone code”, are capable of affecting chromatin structure and gene transcription and are catalyzed by opposing families of enzymes allowing the developmental potential of hematopoietic stem cells to be dynamically regulated [17]. Several genes that are involved in epigenetic modifications are crucial to HSC function, such as the polycomb group gene Bmi1 which has been shown to be critical for long-term maintenance of HSCs in humans [18] and mice [19]. Substitution for H2B-GFP for wild type H2B in these transgenic animals may alter any number of these processes which would then have profound effects on normal HSC function *in vivo*. Also, while reporter genes have been extensively used to monitor a wide variety of biological processes, there is evidence that the presence of the reporter gene may alter the biological activity of certain cells [20], a phenomenon which has been observed in the hematopoietic system [21].

In addition, the inducibility of doxycycline-regulated gene expression in various tissues and cell types strongly depends on the appropriate choice of tetracycline-responsive promoter [22]. Currently available drug-inducible model systems usually rely on the transactivation of artificial promoters by chimeric transcription factors. Although this most often results in high-level transgene expression, the use of native cellular promoters or enhancer elements would be preferable in many circumstances. Moreover, the systems in their present versions are limited by leakiness that results from non-specific binding of the transactivator to the synthetic promoter in the uninduced state. Recent studies have described highly versatile, single vector lentiviral tools that allow for conditional transgene expression from any ubiquitous or tissue-specific promoter as well as for conditional RNA interference [23] and transgenic mice produced by these methods may more allow more accurate lineage tracing than described with the system in this study. Also, the usefulness of GFP as a cell lineage marker has been questioned as potential limitations were highlighted in a recent report comparing three mouse strains in which GFP is considered “ubiquitously expressed”. This study found considerable variation in GFP expression within and between GFP transgenic strains and none of the strains gave truly ubiquitous GFP expression [24]. A detailed analysis of GFP expression in one's tissue of interest must guide the choice of reporter mouse strain when GFP is used as a marker of cell lineage or donor origin.

This technology potentially is a powerful tool for studying potential adult stem cells, or other slow cycling cells, from organs for which few other markers exist, and thus there are currently many efforts to do so using the H2B-GFP mice. However, the studies presented here demonstrate the potential for leaky GFP expression which could prohibit the ability to obtain meaningful data from such studies. The utility of the system is likely to be dependent on several factors, including the mouse strain background, as well as the tissue of study. The effectiveness of the particular promoter-driven tetracycline-regulatable transactivator the H2B-GFP mice are crossed with is likely to have a major impact. It is possible that tet-transactivator transgenes other than the CMV tet-transactivator used here would result in higher expression of the GFP, such that the tet-transactivator-dependent GFP could be distinguished by flow cytometry from the spurious GFP expression demonstrated here that is ∼1 log above background. However, this would have to be clearly demonstrated with appropriate controls, including analysis of GFP expression in the population of interest in mice bearing the H2B-GFP transgene alone. Even so, the two types of GFP expression would be unlikely to be able to be distinguished in tissue sections, since fluorescence levels are far less easily quantified by microscopy. It is likely that generation of new founder lines of H2B-GFP transgenic mice will be necessary for the study of slow-cycling cells in the hematopoietic system. Thus, this study serves as a cautionary tale for use of this potentially useful transgenic mouse line, particularly in the hematopoietic system.

## Materials and Methods

### Mice and Genotyping

H2B-GFP mice were obtained from the Fuchs laboratory (The Rockefeller University, New York). CMV-tTA (TET-off) mice were obtained from Jackson labs (Bar Harbor, Maine). H2B-GFP mice and TET-GFP mice were genotyped by flow cytometric analysis of the peripheral blood with GFP^+^ mice displaying ∼5% GFP^+^ nucleated blood cells. DNA was extracted from tail tips of TET-off and TET-GFP mice using the DirectPCR Lysis Reagent (Viagen Biotech) system and were genotyped with the following PCR primers; CMV-tTA-forward CATGTCCAGATCGAAATCGTC, CMV-tTA-reverse CGCTGTGGGCATTTTACTTTAG. TET-GFP mice were administered doxycycline (2 mg/mL; Sigma) in drinking water supplemented with 5% sucrose and protected from light. The water was changed every three days to ensure activity of doxycycline. All mice were housed in a specific pathogen-free barrier and fed autoclaved acidified water and mouse chow ad libitum were used. All animal procedures were conducted in accordance with the Baylor College of Medicine (Houston, Texas, USA) institutional guidelines.

### Flow Cytometry

For analysis of HSCs, whole bone marrow was collected from the femurs and tibias of mice. Cells were resuspended at 1×10^6^/mL and stained with Hoechst 33342 at 37°C for 90 minutes and subsequently stained with Pe-Cy5 conjugated antibodies against mature lineage markers (Gr-1, Mac-1, CD4, CD8, B220 and Ter119; all from eBioscience) as well as c-Kit-PE and Sca-1-APC (both from BD Pharmingen). HSCs were defined as cells displaying the side population (SP) phenotype, c-Kit^+^Lin^-^Sca-1^+^ (SP^KLS^). Flow cytometric analysis was performed on a triple laser MoFlo (DakoCytomation).

### Histology and Immunofluorescence

Femurs were dissected and fixed by immersion in 4% PFA/PBS for three days followed by three days in 30% sucrose. Bones were mounted in OCT (Tissue-Tek) and snap frozen. Sections were cut on a cryostat and transferred to slides with the Cryojane tape transfer system (Instrumedics). Slides were air-dried for 2 hours and stored at −80°C until use. For immunofluorescence, slides were washed in PBS, permeabilized for 10 minutes in PBTX and blocked for 30 minutes in PBS+1% BSA. Slides were incubated in PBS+1% BSA with rabbit anti-GFP primary antibody (1∶200 dilution; Sigma) for 1 hour at room temperature. Slides were then washed for 30 minutes and then incubated with anti-rabbit Alexa-594 secondary antibody (1∶500 dilution; Molecular Probes) for 1 hour at room temperature. Slides were mounted in Vectashield+DAPI (Vector Laboratories) and images captured on a Zeiss Axioplan 2 microscope equipped with Photometrics Coolsnap HQ camera.

### Competitive Bone Marrow Transplantation

Following a split dose of 10.5 Gy of whole body irradiation, C57Bl/6-CD45.2 recipients (n = 4 for each group) were transplanted via retro-orbital intravenous injection with 200 GFP^+^ or GFP^−^ bone marrow cells from C57Bl/6-CD45.1 TET-GFP mice treated with doxycycline for 12 weeks. Donor cells were competed against 200,000 CD45.2 whole bone marrow cells. Recipient mice were analyzed for donor cell engraftment 12 weeks post-transplant. Following red blood cell lysis, peripheral blood samples were incubated with CD45.1-APC (BD Biosciences), CD4-Pacific Blue, CD8-Pacific Blue, B220-Pacific Blue, B220-PeCy7, Mac-1-PeCy7 and Gr-1-PE antibodies (all eBioscience). Samples were analyzed on a LSRII (BD) flow cytometer.

### HSC Stimulation by 5-Fluoruracil

For 5-Fluorouracil (5-FU) treatment, mice were injected intraperitoneally with a single dose of 5-FU (150 mg/kg body weight; Sigma) and sacrificed 6 or 13 days after injection for HSC analysis.
